# Facilitators, Barriers, and Structural Determinants of Physical Activity in Nulliparous Pregnant Women: A Qualitative Study

**DOI:** 10.1155/2022/5543684

**Published:** 2022-04-11

**Authors:** Leila Kianfard, Shamsaddin Niknami, Farkhonde Amin SHokravi, Sakineh Rakhshanderou

**Affiliations:** ^1^Department of Health Education and Health Promotion, Faculty of Medical Sciences, Tarbiat Modares University, Tehran, Iran; ^2^Department of Health Education and Health Promotion, Faculty of Medical Sciences, Shahid Beheshti University of Medical Sciences, Tehran, Iran

## Abstract

*Aims & Backgrounds*. Reduced physical activity in pregnant women is highly stemmed from their misconceptions and attitudes during pregnancy. This study is aimed at recognizing the facilitators, barriers, and structural factors that influence activity among pregnant women. *Participants & Methods*. This qualitative study was conducted from January to June 2020 in nulliparous pregnant women. Forty participants selected randomly from the Pounak Health Center of Tehran City, Iran, answered open-ended questions about the obstacles that deprived them of physical activity during pregnancy. Data were analyzed by MAXQDA 12 software. *Findings*. 620 primary codes, 42 secondary codes, 11 subthemes, and 6 themes were extracted. These themes were divided into the PEN-3 categories: facilitators, barriers, and structural factors. The nurture factors as facilitators had communication and support from others as subthemes. Barriers consisted of sociocultural (participate in pregnancy class with a companion, social beliefs, and culture of poverty), socioeconomic (financial problems), and individual factors (physical, psychoemotional, and spiritual dimensions), and structural factors consisted of environmental (equipment) and organizational (possibilities in health centers) factors. *Conclusion.* Lack of awareness and misinformation, accessibility obstacles, and economic problems are the worst physical activity barriers during pregnancy. Being among other pregnant women and the physicians' recommendations are the best facilitators of physical activity during pregnancy.

## 1. Introduction

Pregnancy is one of the most sensitive and significant stages of any woman's life. It is associated with changes in psychological needs (e.g., anxiety and depression) and physical demands (such as weight gain and higher cardiac output), and maternal health is the priority of this period [1]. Nevertheless, about 60% of pregnant women eliminate or reduce their physical activity during pregnancy [2, 3].

Researches showed that reduced physical activity in pregnant women is highly stemmed from their misconceptions and attitudes during pregnancy. Regular physical activity in the first six months of pregnancy reduces the prevalence of cesarean section, shortens the second stage of labor, and reduces fetal complications. There are so many pieces of evidence that exercise plays a significant role in preventing diseases, e.g., cancers, type 2 diabetes, obesity, and hypertension [4]. Physical activity during pregnancy has further advantages like a reduced risk of gestational overweight, preeclampsia, and diabetes mellitus due to pregnancy [5].

The prevalence of required standard pregnancy pieces of training in Iran is not satisfactory. This may be important for pregnant women living in low- and middle-income countries (LMICs) because the socioeconomic status (SES) is a risk factor that leads to the prevalence of weight gain, lack of activity, impaired glucose intolerance, and gestational diabetes mellitus (GDM) [6–8].

Physical activity is defined by the World Health Organization (WHO) as “any physical movement produced by skeletal muscle that requires energy consumption, including activities such as working, playing, doing household chores, traveling, and taking responsibility for recreational activities” [9]. Despite the expanding awareness on physiological manifestations of exercise during pregnancy, there is still no comprehensive understanding of the effects of exercise with different intensities and intervals during pregnancy on mother and fetus [10]. According to instructions, it is also recommended that participants need to do exercise for 30 minutes or more and it is better throughout the week [11, 12]. The American College of Obstetrics and Gynecology (ACOG) believes that pregnant women must have physical activity for at least 30 minutes, five days a week [ACOG [13]]. Along with dieting, having activity during pregnancy promises a natural and impressive intervention for infant and maternal health [14, 15]. However, some pregnant women seem not to follow the guidelines [16]; meanwhile, the study in developing countries showed that 70% of pregnant women did not have any physical activity [17].

Having activity is one of the substantial factors affecting the quality of life (QOL). It has increasingly specified that assessing the quality of life can predict the health situation in various groups [18]. The postpartum period can be introduced as an opportunity to change health-related habits throughout life. Unfortunately, limited interventions have been proposed based on health education theories and models in this regard [19, 20] and most studies have been descriptive and analytical. Because pregnant women are not regularly active, researchers should look for barriers, facilitators, and structural factors that prevent exercise during pregnancy. Much research has been done on physical activity and interventions in the United States, Canada, and the Pacific [21]. Clarke & Gross [22] found that pregnant women usually reduce physical activity due to physical limitations, low motivation, and goals or a sense of danger about the activity. Rajabi et al. [23] found that performing regular pregnancy activities may reduce cesarean section.

The PEN-3 cultural model has effectively guided researchers to recognize various affecting factors of health issues [24], and it can be appropriate to use this model as a functional pattern in groups like nulliparous women. This study is aimed at recognizing the facilitators, barriers, and structural factors that influence activity among pregnant women who have not met the guidelines for physical activity in pregnancy.

## 2. Participants and Methods

This qualitative study was conducted from January to June 2020 in nulliparous pregnant women from the Pounak Health Center in the 5^th^ district of Tehran city, Iran, to identify the determinant factors that prevent physical activity during pregnancy. The sampling was performed and continued until the data saturation. Fifty-two pregnant women were eligible according to the following criteria: (i) not having any background disease (hypertension, preeclampsia, eclampsia, heart disease, placenta previa, placenta accrete, etc.), (ii) a prepregnancy body mass index (BMI) equal or higher than 25 (i.e., overweight or obese), (iii) being able to speak and write in Persian, (iv) nulliparous (first pregnancy,), and (v) older than 18 and less than 40 years. After calling the participants, 12 could not be available because they had changed their phone numbers, rejected calls, or lost to response after three messages. The rest of the pregnant women who were successfully called all agreed to participate (Table 1). The study aims were explained to the participants, and all have signed the written informed consent. They were promised data confidentiality and allowed to stop participating at any time they require.

After being approved by the research ethics committee of Tarbiat Modares University, data was collected using in-depth interviews by four trained researchers (three midwives and one psychologist). The researchers used a framework approach to describe and explore the original data concerning the PEN-3 approach [25]. The interviewers set several appointments per week with the participants to decrease their anxiety, and all their experiences and views about physical activity during pregnancy were negotiated, discussed, and recorded precisely. The researchers used a descriptive approach. This approach is appropriate for achieving experiences of pregnant women [26, 27]. This method is very good to speak about topics to get comments that may not be discussed in one-to-one situations such as qualitative and focus groups [28].

Furthermore, in-depth interviews are valuable as health education and health promotion developments, interventions, and contents [29]. The participants were guided with semistructured questions, and answers were compiled to comprehend the investigators' perspectives. This topic focused on services and perceptual and conceptual factors (e.g., knowledge and attitude about physical activity in pregnancy) and participants' communication factors (social networks). Each in-depth interview session lasted about 85 minutes [30, 31]. All the interviews were written, recorded, and transcribed during the conversation. Then, these sessions were guided by a semistructured interview between the participant and the researcher. Some semistructured questions on perceptions, attitudes, facilities, and enabling factors were as follows:

- Which factors do you think lead not to do exercise
- Which factors you think can help in exercise
- Explain the possibilities to exercise in your pregnancy
- What are the motivating factors for attending physical activity classes during pregnancy

All the collected data were entered into MAXQDA 12 software. Codes were sorted and extracted from participants' comments and their states. The participants' quotations were classified into broader themes; after that, themes were unified or changed in a suitable form if needed. The data analysis was argumentative and continuously revised by the researchers. In the end, consensus about themes was reached. The raw data were classified into barriers and facilitator factors to do physical activity in pregnancy. All 40 participants of pregnant women were called for member checking, and data were changed and refined. Finally, the team arranged codes into phrases; after that, analog codes were transpired into subthemes and in the following subthemes that emerged to earn principle themes.

The principal themes were refined and changed through discussions in the interviews to certify that they exactly show the primary data and it was what the pregnant women mentioned in the sessions. Finally, the investigator's process permitted doing the study's internal validity [32]. The qualitative data were reported, and at the end, these are adapted and sorted with RATS (relevance, appropriateness, transparency, and soundness) guideline [33].

### 2.1. Findings

By analyzing the collected data, 620 primary codes, 42 secondary codes, 11 subthemes, and six themes were extracted. According to the PEN-3 cultural model, the participants' opinions about physical activity during pregnancy were categorized into facilitators, barriers, and structural factors (Table 2).

### 2.2. Facilitators

During the interviews with pregnant women, several factors were extracted as facilitating factors. These facilitators were extracted from the interview while answering questions such as “what factors in your pregnancy can help you to do exercise?”

### 2.3. Nurture Factors

It seems that the advice of people who specialize in exercise is very important and necessary for exercising.

“When my doctor recommends that I exercise, I do my best to attend pregnancy classes, but if she does not recommend, I do not go to exercise classes”(participant no.16).

It seemed that talking to other pregnant women and using their experiences encouraged them to do physical activity.

“When I came to the health center for pregnancy care, I saw that other pregnant women talked about exercise classes and became curious and took the address from them. […laughs] This does not seem convenient… At first! [silence]” (participant no. 22).

Communication between pregnant women in social media was effective in encouraging women to do physical activity during pregnancy.

“In this situation, there is a coronavirus disease that I am afraid to leave the house. It is very good to use the Internet to ask my questions to other pregnant women” (participant no. 5).

Awareness about free classes was suggested as an essential motivator for presence in the sports class.

“Stolen water is sweet” [laughs]. She explained that “Since I got pregnant, I lost my job, and my husband's income is not high; therefore, it is motivational for me to participate in these classes” (participant no. 34).

Virtual communication by a group of pregnant women in the same class makes the social motivation stronger. Its traditional method was similar to text message services to motivate them to do physical activity.

“I am encouraged by my friends because umm ... My friends in my group are invited to do physical activity during pregnancy, and when I get bored, I go to social media and talk with my friends. I get excited. The group [...] encourages you to feel as satisfied as to do physical activity with them” (participant no. 12).

When health was not offered as the primary motivation for exercise during pregnancy, pregnant women tended to go to the gym because of their new relationships with other women. Women talked about experiencing reduced back pain during pregnancy, reduced ankle and knee ache, more joy and peace, and increased cardio output.

“Before my pregnancy I had depression, and when I became pregnant unintentionally, my wife and family were anxious about the deterioration of my condition […] Until one of my friends advised me to take part in pregnancy exercises classes, after a few sessions, I am feeling very well In terms of mentally and physically, and I do not use other depressions pills–! [laughs]” (participant no. 3).

### 2.4. Barriers

Barriers to physical activity in pregnant women were specified into three themes: individual, social, and socioeconomic factors.

### 2.5. Sociocultural Factors

Subjective norms decrease the opportunities for doing physical activity. Pregnant women infrequently reported exercise during pregnancy. The investigators scarcely saw the pregnant women exercise with others, but the fit was found that if their husbands were attending pregnancy classes, they would be encouraged to do physical activities. Thirty-two participants said that they would like to participate in exercise classes with other women. Furthermore, 28 participants mentioned the lack of exercise facilities for pregnant women, such as fitness classes. Six women did not agree with men's presence in their pregnant women's exercise classes within follow-up periods.

“My husband and I practically talk about physical activity during pregnancy every day as I like to do exercise, but my husband does not allow me to do that; he thinks it is dangerous for our baby” (participant no. 15)!

Many participants requested that there be someone in the pregnancy class who could teach them pregnancy exercises specifically.

“I would like someone in the sports class to teach pregnancy exercises exclusively to other women” (participant no. 27).

Some pregnant women do not attend pregnancy classes due to the disapproval of other people in the community.

“When I talk to my co-workers about exercising at work, they say it is dangerous for the fetus, and you should not participate” (participant no. 8).

Participants were asked whom they consulted about physical activity, and most of them have said that they try to consult with their doctor for regular exercise.

“I was banned from exercising with other women and their families because I thought exercise is dangerous in this period” (participant no. 36).

Many participants stated that the women often did not support each other due to the low social culture.

“It is hard to explain, but I think that in this country, the culture of pregnant women's sports is still absent, and many families are not allowed to exercise because they are misinformed about it” (participant no. 33).

Many pregnant women claim that people in the community believe that exercising can lead to miscarriage. When asked during frequent visits, some participants confirmed this idea. One of them strongly disagreed with this idea. This is the main effect of encouraging them to be active during pregnancy. Among the 12 women who explained that pregnant women were encouraged to exercise, eight did not. A small number of participants said that if they had a spouse who could encourage them to attend classes, they would probably do physical activity. Several participants had not confirmed the role of their extended family, whereas a few did.

### 2.6. Socioeconomic Factors

Socioeconomic factors do not allow participants to take part freely in exercise classes. Within interviews, they reported a variety of socioeconomic restrictions. First, women said that there is no proper place to do exercise. Several participants noted that homes are small and not suitable for doing physical activity. Some women said that being active at home and doing activities such as washing and cleaning are enough exercise in their pregnancy.

“I prefer to select a sports class that is not expensive that I could have a private trainer. If physical activity classes were held for pregnant women in health centers or were given free cards to participate in exercise classes, they would use more…” (participant no. 2).

One participant described the inactivity during her pregnancy:

“The city is very crowded, and everywhere in the city is built houses, and there is not even the right place to walk for pregnant women, especially with this polluted air … no… in this daytime is no way! It is a place, not for walking” (participant no. 9)!

One pregnant woman commented that

“I do not have to pay for my diet, but if I want to attend pregnancy classes, I have to pay. Because of this, I think it is better than having a gym for health. It is not expensive, so I think the diet has more effectiveness than physical activity” (participant no. 18).

Many pregnant women believe that because of economic sanctions, the cost of classes is getting more expensive every day and they can not afford the heavy costs.

“My husband works hard, and I can not tell him that the cost of pregnancy classes has increased and I have to cancel going to class” (participant no. 11).

During the interviews, 18 women spoke of their spouse's economic situation and claimed that they were not allowed to attend exercise classes because of their financial problems and they did not get awareness about free sports classes for participating. This means that health care providers must provide all information about these classes' existence to pregnant women and social networks and media must inform them.

Most women, who do not have enough money or time to attend classes, try to be active. Several of these participants described a diversity of main strategies, including having mobility, dancing, walking, and jobs that need activity at home. However, some women encountered structural problems (husbands' opposition or family, affordable classes) that confined them to classes or mobility. It seems that outdoor is not a suitable place for pregnant women. For one of the participants, walking was a comfortable form of physical activity because of financial constraints.

“Walking to the shopping center and reported: Instead of sitting in the car for a long time, I prefer only to walk” (participant no. 34).

### 2.7. Individual Factors

Many women said that they did not know how to attend sports classes in hospitals or health centers. This lack of awareness can be due to the neglect of healthcare personnel who do not inform women of their visits or have educational or social media weaknesses. Mother's health is not the primary motivation for exercising during pregnancy. One woman summarized this and said that

“I would rather eat less than exercise and get tired, but if it does not work, it is too hard and bad because of my body condition, and it is not good at all” (participant no. 5).

However, few pregnant women said that if they feel the risk of having pregnancy-induced diabetes due to a history of familial or high blood pressure, which seriously danger their fetal deaths, they try to participate in exercise classes.

“My baby's health is more important than myself, but I am apprehensive about diabetes and hypertension due to my obesity in pregnancy, and I prefer to exercise for my health. Things just happen…” (participant no. 27).

One woman commented that the old opinion and belief confined the culture:

“Previously, women had much activity and therefore was easier to give birth or had any problems, but with the advancement of technology, nobody cares about this issue” (participant no. 31).

Many women believed that they would no longer need exercise if they could control their weight through healthy nutrition.

“I always had my health rights before pregnancy, and I was very active, but now due to my baby not being injured, I prefer to take a diet and do not let the illness come to me to have a more comfortable delivery” (participant no. 10).

The belief in pregnant women that having healthy eating is more comfortable and more effective than doing physical activity during pregnancy reflects the health professionals' role in increasing women's awareness and cultural poverty in this community.

“In my opinion that everything is dependent on nutritional health. If pregnant women regard and take care of their diet, I am sure that they do not get overweight and obesity and no longer need to exercise” (participant no. 14).

Another woman described that

“Maintaining a healthy diet avoids exercise during pregnancy and reduces the risk of harm to the baby” (participant no. 5).

Almost all women who participated in the interview agreed that healthy food would reduce the need for physical activity in pregnancy. Just seven women recognized fitness as the first motivation to do exercise in pregnancy.

“Whenever I go to my doctor's or healthcare provider's visit, she never advises me to exercise for my health” (participant no. 13).

Therefore, most women thought about physical activity as fitness and did not know what benefits exercise has during pregnancy.

“Nutrition advisor recommended me that I could lose more than 10 pounds if I remained on a severe diet” (participant no. 40).

## 3. Structural Factors

### 3.1. Environmental Factor

Some participants said dancing and stretching movement are a good alternative for physical activity in pregnancy; there is no need to spend time or money outdoors. They did describe dancing as not requiring space or individual facilities or paying cash, and they saw dancing as fun.

“I provided the device for myself at home, and I do not like to get out of the house due to air pollution, and I try to exercise at home so that we do not face any problems because of the pregnancy situation. –However, I do not like to do that for a long time” (participant no. 7).

One woman commented that

“I think dancing and aerobic instead of exercising is much better. I do not like to do slow movements” (participant no. 1).

### 3.2. Organizational Factors

Some pregnant women mentioned that working out of home is like physical activity.

“I work in a hospital as a nurse. My work requires a lot of energy, and I am always moving, and it is a kind of physical activity, especially during pregnancy. I do not like to do more activities after leaving work because I am afraid it will be dangerous for my baby” (participant no. 16).

Employed women with sedentary and nonmobile jobs said that they would like to be active and have the opportunity to exercise.

“For instance, if I have no time to do physical activity at my desk, I am trying to walk around in my workplace […] my office is so far from my work and walk to there every day. Like… about ten minutes” (participant no. 21).

Another woman, who worked in an office, said that

“Due to pregnancy, someone does not let me work at home, but I always try to be active” (participant no. 13).

Another woman described the cost of pregnancy classes as a significant barrier to pregnancy:

“Exercising at a gym class near her workplace, which costs $10 per week. Another woman described close to my workplace; due to my pregnancy situation and economic problems, I found the gym class that is also affordable for me” (participant no. 33).

## 4. Discussion

This qualitative study explored barriers, facilitators, structural factors, and possibly other stimuli during pregnancy that influenced physical activity. At first, subjective norms constrain pregnant women from being active and participating in exercise classes. By accepting the PEN-3 approach, we have extracted a range of barriers, facilitators, and structural factors that affect pregnant women's intentions to be active during pregnancy. The interviewers distanced from the expert's role and pretended that they were only interested in participants' perceptions and beliefs and emphasized that there were no right or wrong answers during the interview.

Most pregnant women stated that they were encouraged to exercise only in the presence of other pregnant women, but few mentioned the need to be with their husbands. Employed women had limited time to exercise, and some had financial difficulties attending sports classes. Women with a first pregnancy faced a diversity of obstacles related to socioeconomic situations. Pregnant women that live in a house with the deep space limited to have opportunities to do housing exercise and participation in sports classes were imagined; it would be expensive for women with low income.

Interestingly, spouses and extended family members are described as barriers to exercise in pregnant women, indicating the power of cultural influence in developing countries and thus show the need to increase awareness of health literacy in the society. Finally, some women described that friends', husbands', and physicians' recommendations to do physical activity are useful. Few women bolded the advantage of weight loss on other health concerns; also, they were more concerned about their infant's health, which shows the need to raise awareness in women at the community level. Some participants described healthy nutrition during their pregnancy as a kind and suitable replacement for being active in this period. According to the results, many factors seemed to motivate physical activity during pregnancy, e.g., social support and nurtures, which are very useful in developing countries, presenting further motivation for consistency.

Many pregnant women with financial difficulties were looking for low-cost classes. Health as a primary motivation was scarcely discussed for doing physical activity in this period. Participants discussed unwillingness to attend pregnancy exercise classes; for instance, talk about fitness, which was the essential and primary motivation in this group. Although the findings of this qualitative study about facilitators and barriers and structural factors' effects around physical activity during pregnancy are available in developing countries, prominent elaborations that may be more bolded in scary resourced bases are yet missing in qualitative studies about exercise [34, 35]. Some studies have shown that fitness and preventing overweight during pregnancy have a solid motivation to exercise [36, 37].

Although many pregnant women have suggested dieting to prevent weight gain to exercise during pregnancy, the scientific evidence of their experience shows that physical activity is necessary for dramatic weight loss [38]. One intervention study has explained that weight loss significantly happened in pregnant women who walked almost 75 minutes per day instead of the guideline that recommended having 30 minutes of activity per day [35]. This suggests that women are more likely to consider their infants' health than their weight loss and fitness during pregnancy. This point of view may show the absence of physical activity for weight gain in developing countries. However, there is evidence that pregnancy overweight is accommodation with detrimental results and side effects for pregnant women or their infants [36]. Thus, it is necessary to design other approaches to weight loss and exercise interventions during pregnancy.

In our study, some pregnant women demonstrated their husbands as a significant obstacle to maintain physical activity. Conducting classes for pregnant women with their spouses may be practical and advantageous at decreasing social dissociation.

Economic community-based physical activity classes, sanctions, and being away from sports classes from the resident place can explain many barriers to physical activity in pregnancy. Trying to increase facilities and the number of classes for easier access could be a way to create opportunities for pregnant women to do physical activity. Social support was shown to be an obstacle to being active in first-pregnant women [39]. More financial and time aspects relate to the unemployed or those whose husbands have low incomes [39]. One of the problems encountered by many women is the ability to perform the exercise in pregnancy and that women's awareness of opportunities was deficient. This reflects the full role of physicians, midwives, and healthcare providers in informing them. In previous studies, healthcare providers provided helpful advice and information to pregnant women about physical activity and a healthy diet [40]. In other studies, it has been shown that many employed women, who do not have enough time to be active, have stated that the same work they do is enough to exercise pregnancy [39]. Many women cannot participate in pregnancy classes in developing countries due to air pollution and traffic problems [40, 41]. The critical issue raised by participants in this study is the lack of interest of pregnant women in pregnancy exercise, which they assume can lead to irreparable harm to their babies, and therefore, they do not want to attend these courses. Therefore, most of them preferred to control their weight by diet. These findings may be impressed by the results that pregnant women have received of the conflicting messages during this period.

This study also faced many restrictions. It was specifically concentrated on pregnant women, but the research about husbands' and physicians' attitudes, perceptions, nurtures, and behaviors can also help to determine the reasons for physical inactivity during pregnancy. Besides, not all the investigators in the first interview participated in the second in-depth interview. The age of participants (18 to 40 years old) was another restriction of this study. However, older participants may have very different experiences and may face several obstacles. Besides, girls who are in puberty mostly spend less time on physical activity. Unfortunately, we do not have access to this group range in health centers [42].

## 5. Conclusion

Lack of awareness and misinformation, accessibility obstacles, and economic problems are the worst physical activity barriers during pregnancy. Being among other pregnant women and the physicians' recommendations are the best facilitators of physical activity during pregnancy.

## Figures and Tables

**Table 1:** Participant characteristics (*n* = 40).

*General*	
Average age	30.5 ± 6.0 years (18–38)
Average BMI	29.2 ± 7.7 (25–51)
Gestation (wk)	26.4 ± 3.7
Prepregnancy body mass (kg)	65.8 ± 12.5
*Education*	
Secondary school completed	8 (20%)
Technical/secretarial after secondary school	16 (40%)
College/university completed	15 (37/5%)
Postgraduate degree	1 (2/5%)
*Occupation*	
High energy demanded	6 (15%)
Intermediate energy demanded	15 (37/5%)
Low energy demanded	11 (27/5%)
Not working	8 (20%)

**Table 2:** The theme, subthemes, and codes of the facilitators, barriers, and structural factors.

Themes	Subthemes		Secondary codes
*Facilitators*			
Nurture factors	Communication		(i) The role of the physician to advise to do physical activity (ii) The support of other pregnant women to motivate each other to do physical activity (iii) Conversation among pregnant women in the virtual network
	Support from others		(i) The role of the husband to motivate to do physical activity during pregnancy (ii) The purpose of friends to support the woman (iii) The critical role of the family to motivate pregnant women
*Barriers*			
Sociocultural factors	Participate in pregnancy class with a companion		(i) Attendance of the spouse as a companion in participating in pregnancy sports classes (ii) Having other women in the pregnancy class motivates me to attend pregnancy classes (iii) Having a dedicated sports instructor motivates me to take pregnancy classes
	Social beliefs		(i) Society believes that exercise is dangerous during pregnancy and harmful to the fetus (ii) Public opinion on the dangers of pregnancy exercise for the fetus (iii) Lack of community approval for pregnant women to be active during pregnancy
	Culture of poverty		(i) Lack of support from the family's motivation to do physical activity during pregnancy (ii) Weak family culture to encourage pregnant women to exercise (iii) Lack of correct view of the usefulness of pregnant women during pregnancy to have a healthy child
Socioeconomic factors	Financial problems		(i) Low income to participate in the classes (ii) Expensive pregnancy exercise classes (iii)Lack of price stability due to economic sanctions
Individual factors	Physical dimension	Physiological condition	(i) Overweight and heaviness in pregnancy (ii) Difficulty in breathing during pregnancy (iii) Physiological changes in the body during pregnancy
		Pathological condition	(i) Difficulty exercising during pregnancy (ii) Physical problems due to pregnancy (iii) Having an underlying disease and fear of getting worse
	Psychoemotional dimension	Attitude	(i) Believing that exercise is not useful during pregnancy (ii) Believing that obesity does not affect fetal health (iii) Believing that exercise is dangerous in pregnancy for the mother and fetus
		Primary and secondary reactions	(i) Compliance with the disease (ii) Inability to adapt to pregnancy changes (iii) Uncontrolled diseases caused by pregnancy
	Spiritual dimension		(i) Negative thinking (ii) Fear of harm to the fetus (iii) Stress due to the lack of support from pregnant women (iv) Lack of confidence in having the ability to do exercise
*Structural factors*			
Environmental factors	Equipment		(i) Lack of sports space to participate in classes (ii) Lack of sports place to participate in classes (iii) Lack of proper facilities in the exercise class for pregnant women (iv) Lack of proper placards to inform the community
Organizational factors	Possibilities in health centers		(i) Lack of professional staff in health-treatment centers (ii) Lack of sufficient information among health center staff (iii) Lack of a specialist in health centers for training (iv) Lack of suitable places in health centers for holding pregnancy sports classes

## Data Availability

All data generated during the process of this research are included in this article.
